# Construction of Novel lncRNA–miRNA–mRNA Network Associated With Recurrence and Identification of Immune-Related Potential Regulatory Axis in Hepatocellular Carcinoma

**DOI:** 10.3389/fonc.2021.626663

**Published:** 2021-07-15

**Authors:** Tian Zhan, Xiang Gao, Guoguang Wang, Fan Li, Jian Shen, Chen Lu, Lei Xu, Yuan Li, Jianping Zhang

**Affiliations:** ^1^ Department of General Surgery, The Second Affiliated Hospital, Nanjing Medical University, Nanjing, China; ^2^ The Collaborative Innovation Center for Cancer Personalized Medicine, School of Public Health, Nanjing Medical University, Nanjing, China

**Keywords:** lncRNA-miRNA-mRNA network, recurrence, HCC, ASF1B, immune infiltration

## Abstract

Hepatocellular carcinoma (HCC) is one of the most common malignant diseases globally. Despite continuous improvement of treatment methods, high postoperative recurrence rate remains an urgent problem. In order to determine the mechanism underlying recurrence of liver cancer and identify prognostic genes, data from the Gene Expression Omnibus (GEO) and The Cancer Genome Atlas (TCGA) were integrated and analyzed. Differentially expressed genes (DEGs) between HCC tissue and normal liver tissue were identified, and a protein–protein interaction network was constructed to find hub genes. Clinical correlation analysis and disease-free survival (DFS) analysis were performed using the R language and GEPIA to identify relapse-related genes. Correlation analysis was used to identify a potential regulatory axis. Dual-luciferase reporter gene assay was used to confirm the reliability of the long non-coding RNA (lncRNA)–microRNA (miRNA)–mRNA regulatory axis. Immune infiltration analysis was performed using the TIMER database. Correlations between immune gene markers and ASF1B were verified using quantitative real-time polymerase chain reaction (RT-qPCR). In this work, we found that nine lncRNAs and five mRNAs were significantly overexpressed in HCC tissues from patients with recurrence. *SNHG3*, *LINC00205*, *ASF1B*, *AURKB*, *CCNB1*, *CDKN3*, and *DTL* were also closely related to HCC grade and stage. Survival analysis showed that these seven DEGs were significantly correlated with poor DFS. Correlation analysis identified *SNHG3*–*miR-214-3p*–*ASF1B* as a potential regulatory axis. Dual-luciferase reporter gene assay showed that SNHG3 and ASF1B directly bound to miR-214-3p. ASF1B was negatively regulated by miRNA-214-3p, and overexpression of SNHG3 could inhibit the expression of miRNA-214-3p. In addition, *ASF1B* was positively correlated with immune infiltration. A reduction in ASF1B could markedly inhibit the expression of CD86, CD8, STAT1, STAT4, CD68, and PD1 in HCC cells. Flow cytometry showed that SNHG3 promoted the PD-1 expression by regulating ASF1B. Meanwhile, elevated *ASF1B* predicted poor prognosis of HCC patients in subgroups with decreased B cells, CD8+ T cells, or neutrophils, and those with enriched CD4+ T cells. In conclusion, we found that a novel lncRNA SNHG3/miR-214-3p/ASF1B axis could promote the recurrence of HCC by regulating immune infiltration.

## Introduction

Liver cancer is the second leading cause of tumor-related deaths in men globally, especially in developing countries. There was an estimated 782,500 new cases of liver cancer and 745,500 deaths globally in 2012, of which China accounted for about 50% (1). Hepatocellular carcinoma (HCC) is the most common histological type of liver cancer (2). Hepatitis B virus and hepatitis C virus infection, excessive alcohol consumption, obesity, and cirrhosis of the liver are regarded as risk factors for HCC (3–5). Currently, surgery is the most important treatment strategy. Tumor recurrence is the main cause of death after radical resection of HCC (6). However, although surgical techniques and treatments have improved in recent years, the recurrence rate of HCC remains at 50–70% (7).

At present, serum biomarker Alpha fetoprotein (AFP), vascular invasion, and tumor stage are used to predict the recurrence and prognosis of HCC (8); however, these forecasting methods are based on clinical characteristics and do not consider the complex molecular pathogenesis and biological mechanisms involved in HCC. Moreover, the predictive ability of these methods is not satisfactory (9). Therefore, there is an urgent need to identify new biomarkers to predict tumor recurrence and to better understand its mechanism.

The liver is the largest immune organ in the body and is rich in various immune cells. A recent report revealed that immune tolerance and escape have an important role in the progression of HCC (10). Moreover, previous reports have shown that intratumoral CD3+ and CD8+ T cells are associated with recurrence and overall survival in HCC and colorectal cancer (8, 11). Accumulating evidence indicates that imbalances of pro-inflammatory and anti-inflammatory cells in the tumor microenvironment promote the progression of tumors (12, 13). Therefore, identification of differentially expressed genes (DEGs) related to immune infiltration in cancer will help to improve therapeutic strategies.

In the present study, based on an integrated analysis of four datasets – GSE69164, GSE77509, GSE76903, and The Cancer Genome Atlas (TCGA)-Liver Hepatocellular Carcinoma (LIHC) – we found that patients with poor prognosis of HCC and recurrent carcinoma tended to have high expression of small nucleolar RNA host gene 3 (*SNHG3*), long intergenic non-protein coding RNA 205 (*LINC00205*), anti-silencing function 1B histone chaperone (*ASF1B*), aurora kinase B (*AURKB*), cyclin B1 (*CCNB1*), cyclin-dependent kinase inhibitor 3 (*CDKN3*), and denticleless E3 ubiquitin protein ligase homolog (*DTL*). We also constructed a novel long non-coding RNA (lncRNA)–microRNA (miRNA)–mRNA network related to HCC recurrence. Furthermore, we found that *ASF1B* was correlated with immune cell infiltration, and identified *SNHG3*–*miR-214-3p*–*ASF1B* as an effective regulatory axis. Further analysis indicated that immunotherapy might reduce the risk of postoperative recurrence in HCC *via* effects on *ASF1B*.

## Materials and Methods

### Research Data Sources

In the present study, two mRNA gene expression datasets (GSE69164 and GSE77509) and one miRNA microarray dataset (GSE76903) were obtained from the Gene Expression Omnibus (GEO; http://www.ncbi.nlm.nih.gov/geo), an important database that contains records from chips, as well as second-generation sequencing and other high-throughput sequencing data. The raw microarray count data from the GEO datasets were normalized by a size factor, and probes were translated to the corresponding gene symbol based on platform annotation information. GSE69164 contains 11 HCC tissue samples and 11 adjacent normal liver tissue samples. GSE77509 and GSE76903 contain 20 HCC tissue samples and 20 adjacent normal liver tissue samples. RNA sequencing raw count data, containing lncRNA sequencing data (from 375 HCC tissues and 49 adjacent normal liver tissues), mRNA sequencing matrix data (from 375 HCC tissues and 49 adjacent normal liver tissues), and miRNA sequencing expression results (from 372 HCC tissues and 50 adjacent normal liver tissues), together with clinical information for 309 HCC patients, were downloaded from TCGA-LIHC (https://portal.gdc.cancer.gov/). Our research conforms fully with TCGA and GEO publication requirements; no ethics committee approval was needed.

### Identification of DEGs

The “DESeq2” and “limma” R packages for difference analysis were used in our study. “DESeq2” package applies to RNA sequencing raw count data and only accept integers, and “limma” R package is mainly used for difference analysis of chip data with small sample sizes. In addition, it is generally considered that RNA sequencing raw count data do not conform to a normal distribution, while chip data do; thus, difference analysis using the “limma” package results in large errors for RNA sequencing count data. Therefore, the differentially expressed lncRNAs (DELs) and differentially expressed mRNAs (DEMs) between HCC tissues and adjacent normal liver tissues in TCGA-LIHC were identified using the “DESeq2” package. The screening criteria included two conditions: a false discovery rate (FDR)<0.01 and |log2 fold change (log2 FC)|≥2. The “limma” package was used to identify DEMs and differentially expressed miRNAs (DEMIs) between normal and HCC tissues in the GSE69164, GSE77509, and GSE76903 datasets. The screening criteria for DEMs were |log2 FC|≥2 and adjacent P-value (adj. P)<0.01. For DEMIs, |log2 FC|≥1.5 and adj. P<0.05 were considered to indicate statistical significance.

### Construction of a Preliminary Competing Endogenous RNA (ceRNA) Network

Overlapping mRNAs were selected as DEMs from the GSE69164, GSE77509, and TCGA-LIHC datasets. miRTarBase (http://mirtarbase.cuhk.edu.cn/php/index.php) (14), a database of experimentally validated miRNA–target interactions, was used to predict miRNA–mRNA interactions based on DEMIs from GSE76903. The target mRNAs were intersected with DEMs. Then, we used the starBase database (http://starbase.sysu.edu.cn/) (15) to identify the interactions between miRNAs and lncRNAs. The predicted target lncRNAs were intersected with DELs from the TCGA-LIHC dataset. Based on the interactions of these DEGs, we constructed a preliminary and unverified lncRNA–miRNA–mRNA regulatory network associated with HCC, which was visualized using the Cytoscape software (version 3.6.1).

### Protein–Protein Interaction (PPI) Network Construction and Hub Gene Selection

PPI networks, which reflect the functional interactions between proteins, are used for the identification of key genes in disease generation and development. In this study, a PPI network was constructed using the STRING (Search Tool for the Retrieval of Interacting Genes; http://string-db.org) (version 11.0) online database. An interaction with a combined score ≥0.4 was considered statistically significant. The PPI network was visualized using Cytoscape version 3.6.1. Nodes of degree ≥35 in the PPI network were selected as hub genes. In addition, a ceRNA sub-network based on hub genes was screened from the whole ceRNA network, and corresponding interactions were visualized with Cytoscape.

### Identification of Relapse-Related Genes

To identify relapse-related genes, we first compared expression levels of DEGs between HCC patients with and without recurrence within 3 years. Boxplots were drawn using the “beeswarm” R package. Subsequently, correlations between the expression of DEGs and clinical features (tumor grade and stage) were analyzed based on the clinical information of 309 HCC patients from TCGA. Disease-free survival (DFS) curves were plotted based on the log-rank test using GEPIA (Gene Expression Profiling Interactive Analysis; http://gepia.cancer-pku.cn/) (16), a website for analyzing RNA sequencing data in TPM format from TCGA and the GTEx project.

### Screening of Relapse-Related lncRNA–miRNA–mRNA Network

Correlation analysis was performed to identify a relapse-related lncRNA–miRNA–mRNA network using RNA sequencing data in FPKM format from TCGA-LIHC. The Pearson method was used to determine correlation coefficients. P<0.05 was considered statistically significant. In addition, the expression levels of DEMIs between HCC tissue and normal liver tissue in the network were verified in the TCGA-LIHC dataset. The final relapse-related lncRNA–miRNA–mRNA network was visualized using Cytoscape (17).

### Cell Culture

Two HCC cell lines, HepG2 and HuH-7, were obtained from the American Type Culture Collection (ATCC). All cells were cultivated in Dulbecco’s Modified Eagle’s Medium (DMEM; Gibco, USA) containing 10% fetal bovine serum (FBS; Gibco, USA) in an incubator containing 5% CO_2_ at a constant temperature of 37°C.

### Cell Transfection

HCC cells were inoculated in six-well plates. Seed cells to be 70–90% confluent at transfection according to the instructions of Lipofectamine 3000 (Invitrogen, USA). The transfection plasmids used in this experiment included short interfering RNA (siRNA)-ASF1B, a siRNA negative control (NC), miR-214-3p mimics, NC mimics and SNHG3 overexpression plasmid (SNHG3 pcDNA). Cells were incubated for 48 h after transfection. Transfection efficiency was examined through the subsequent qRT-PCR.

### Quantitative Real-Time Polymerase Chain Reaction (qRT-PCR)

We first used the TRIzol method (Invitrogen, Carlsbad, CA, USA) to extract total RNA from cells. Extracted RNA was reverse transcribed into complementary DNA (cDNA) for qRT-PCR. Glyceraldehyde 3-phosphate dehydrogenase (GAPDH) and U6 were used as endogenous controls. The qRT-PCR experiments followed the instructions of the CHAMQ SYBR qPCR Master Mix kit (Vazyme, Nanjing, China). The primer sequences used in this study are shown in **Table S1**. The mRNA expression levels were analyzed by the 2^-ΔΔCt^ method (18).

### Dual-Luciferase Reporter Gene Assay

The binding sites of miRNA-214-3p in SNHG3 and ASF1B sequences were first predicted using the starBase platform and then expanded by PCR. Subsequently, the amplified fragment was inserted into a vector to construct wild-type (WT) ASF1B (5′-ag**UGCCUGUC**AAG**G**C**U**CCA**G**U**CCUGCUG**a-3′) and WT miRNA-214-3p (3′-ugacggaca**GA**CA**CGGACGAC**a-5′) plasmids. Then, we altered partial nucleotides by a gene mutation technique and constructed mutant-type (MT) ASF1B (5′-ag**ACGGACAG**AAG**C**C**A**CCA**C**U**GGACGAC**a-3′) and MT miRNA-214-3p (3′-ugacggaca**CU**CA**GCCUGCUG**a-5′) plasmids. Cells were co-transfected with the ASF1B-WT or ASF1B-MT plasmid and miR-214-3p mimics, and with the miRNA-214-WT or miRNA-214-MT plasmid and SNHG3 overexpression plasmid. Finally, the luciferase activities in each group were detected.

### Tumor Immune Infiltration Analysis

TIMER is a comprehensive database for analyzing the levels of immune infiltrates in different tumors (https://cistrome.shinyapps.io/timer/) (19). Correlations between relapse-related genes and infiltration levels of different immune cells were estimated using TIMER. Then, the correlations of ASF1B with gene markers of B cells, CD8+ T cells, neutrophils, macrophages, dendritic cells, monocytes, natural killer cells, and regulatory T (Treg) cells in HCC were verified, and tumor purity and patient age were used to correct the P-values. Subsequently, to further verify the relationship between ASF1B and the tumor immune microenvironment, we tested the expressions of partial immune gene markers (COR>0.3 after purity correction) in ASF1B-knockout HCC cells by RT-qPCR.

### Flow Cytometry

The HCC cells were divided into 3 groups. The first group was that a blank vector plasmid control. The second group was that SNHG3 was overexpressed in HepG2 cell. The final group was that HepG2 cells were co-transfected with overexpressed plasmid targeted SNHG3 and knockdown plasmid targeted ASF1B. All Cells were incubated for 72 h after transfection. Then, the cells were harvested and incubated with anti-human CD279 (PD-1) Monoclonal Antibody (MIH4) linked with phycoerythrin (PE) fluorochrome (12-9969-42; eBioscience: Thermo Fisher Scientific, Inc.) for 30 min at 4°C in dark. Cells were resuspended in 0.5 ml phosphate buffer saline (PBS) for flow cytometry.

### Statistical Analysis

GraphPad Prism (version 8.0) and R statistical software (version 3.6.1) were used for statistical analysis. Experimental data were expressed as mean ± standard deviation. The standard t-test was used to compare the differences between the two groups. Correlation analysis was performed using the Spearman rank correlation test. P<0.05 was considered statistically significant.

## Results

### Identification of DEGs in HCC

**Figure 1:**
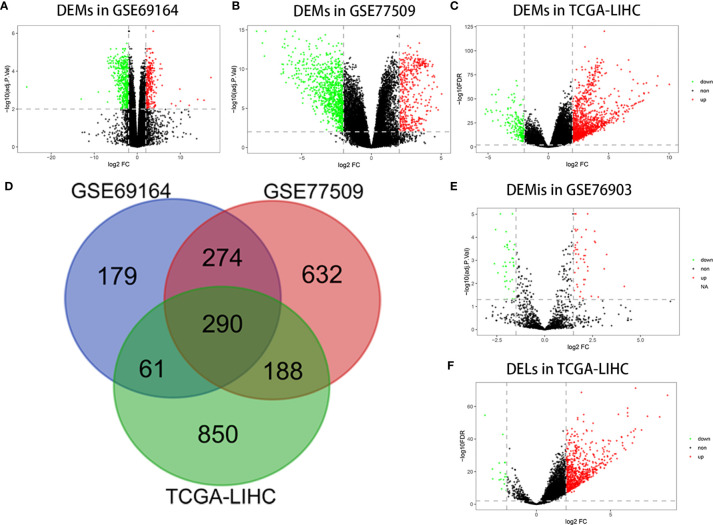
Differentially expressed RNAs analysis. **(A)** DEMs in GSE69164 identified using the limma package. **(B)** DEMs in GSE77509 identified using the limma package. **(C)** DEMs in TCGA-LIHC identified using the DESeq2 package. **(D)** Overlapping DEMs. **(E)** DEMis in GSE76903 identified using the limma package. **(F)** DELs in TCGA-LIHC identified using the DESeq2 package.

A total of 804, 1384, and 1389 mRNAs were screened from the GSE69164, GSE77509, and TCGA-LIHC datasets, respectively, according to the criteria |log2 FC|≥2 and FDR ≤ 0.01 or adj.P<0.01. The 290 overlapping mRNAs among the three datasets were considered to be DEMs. Similarly, 707 upregulated and 16 downregulated DELs were identified from TCGA-LIHC based on the above procedure, as well as 44 upregulated and 38 downregulated DEMIs in GSE76903 based on the criteria of |log2 FC|≥1.5 and adj.P<0.05. Volcano plots of DEMs, DEMIs, and DELs, and a Venn diagram of DEM intersection are shown in **Figure 1**.

### Construction of a Preliminary ceRNA Network

We employed 290 DEMs, 82 DEMIs, and 723 DELs to build a ceRNA network. The lncRNA–miRNA and miRNA–mRNA interactions were predicted based on DEMIs using the starBase and MiRTarBase online databases. Finally, a preliminary and unverified ceRNA network was constructed from 124 lncRNAs, 35 miRNAs, and 80 mRNAs (**Figure S1**). There were 314 connections between lncRNAs and miRNAs, and 152 connections between miRNAs and mRNAs in this network.

### PPI Network Construction and Hub Gene Selection and Analysis

**Figure 2:**
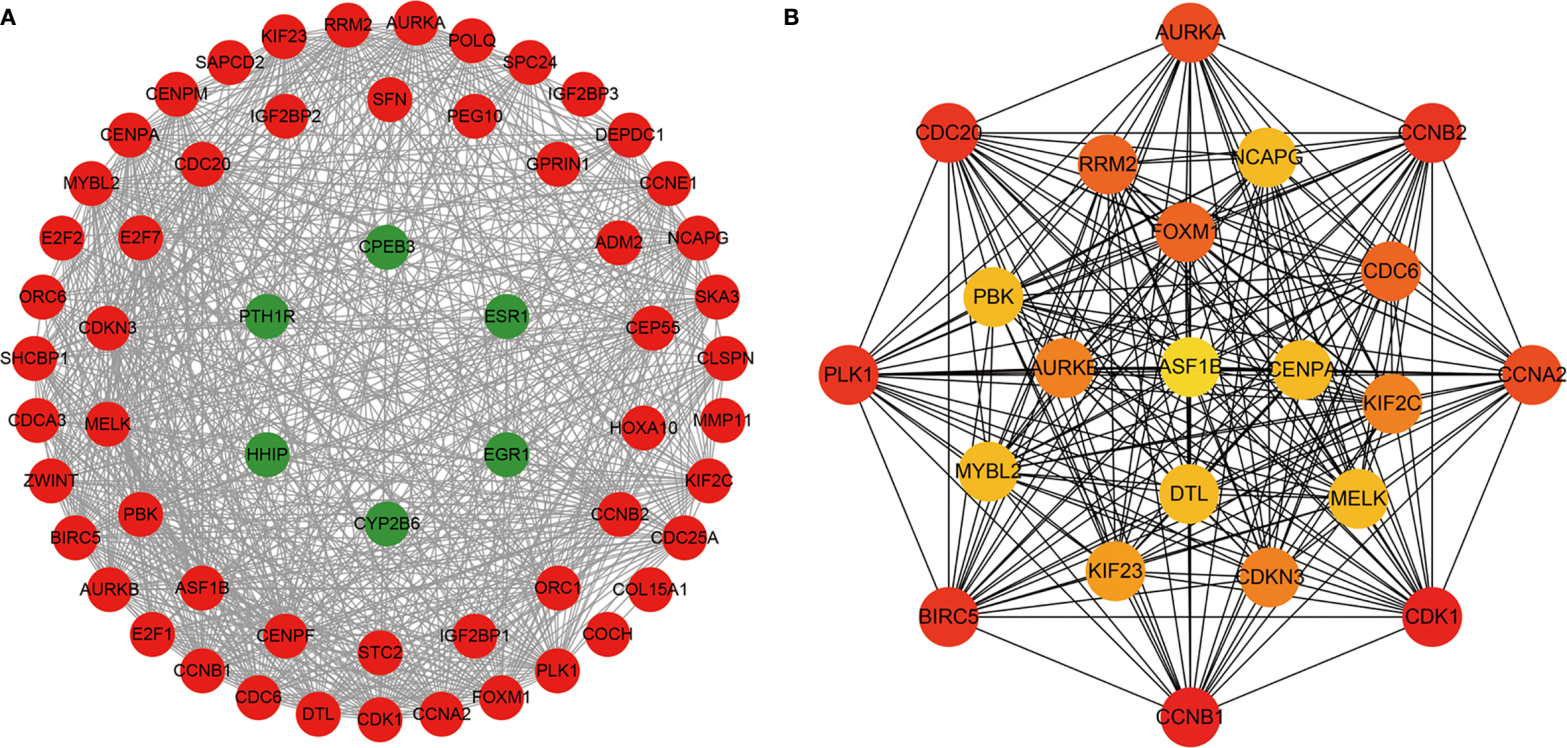
The protein-protein interaction (PPI) network of ceRNA network-related DEMs. **(A)** The nodes denote DEMs (confidence score > 0.4) and red represent up-regulated, green represent down-regulated. **(B)** The hub genes with degree ≥ 35.

To determine the interactions between ceRNA-related mRNAs and explore their functions at the protein level, a PPI network was constructed using the STRING database (**Figure 2A**). The PPI network consisted of 59 nodes and 702 edges. No interactions were found for 21 mRNAs in the PPI network, implying that these mRNAs might be unimportant in the development of HCC; thus, we discarded these 21 mRNAs. A total of 22 genes were considered as hub genes with degree ≥35, and their interactions were obtained using Cytoscape for further study (**Figure 2B**).

### Identification of Relapse-Related DEMs

**Figure 3:**
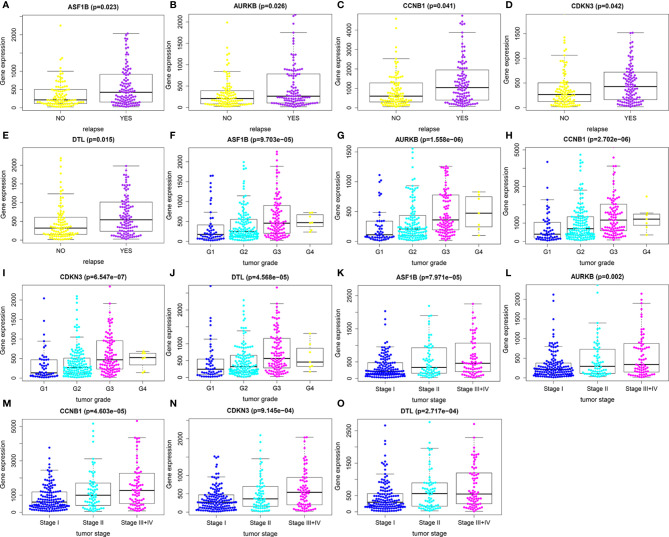
Identification of relapse-related DEMs. **(A–E)** The relationships between expression levels of hub genes and HCC recurrence. **(F–J)** The relationships between expression levels of hub genes and tumor grade. **(K–O)** The relationships of expression levels of hub genes and tumor stage.

**Figure 4:**
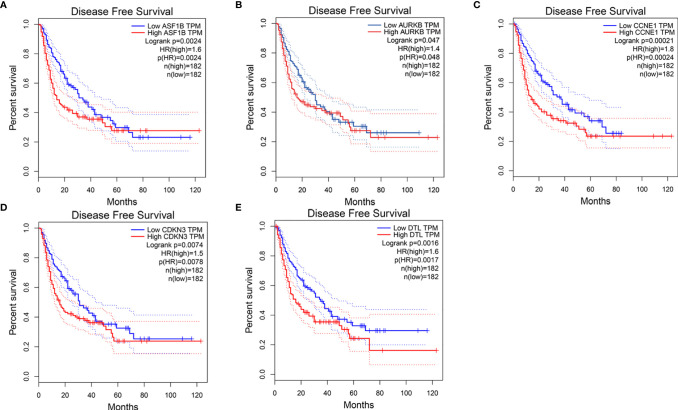
Disease-free survival analysis of **(A)** ASF1B, **(B)** AURKB, **(C)** CCNE1, **(D)** CDKN3, **(E)** DTL by using GEPIA.

**Figure 5:**
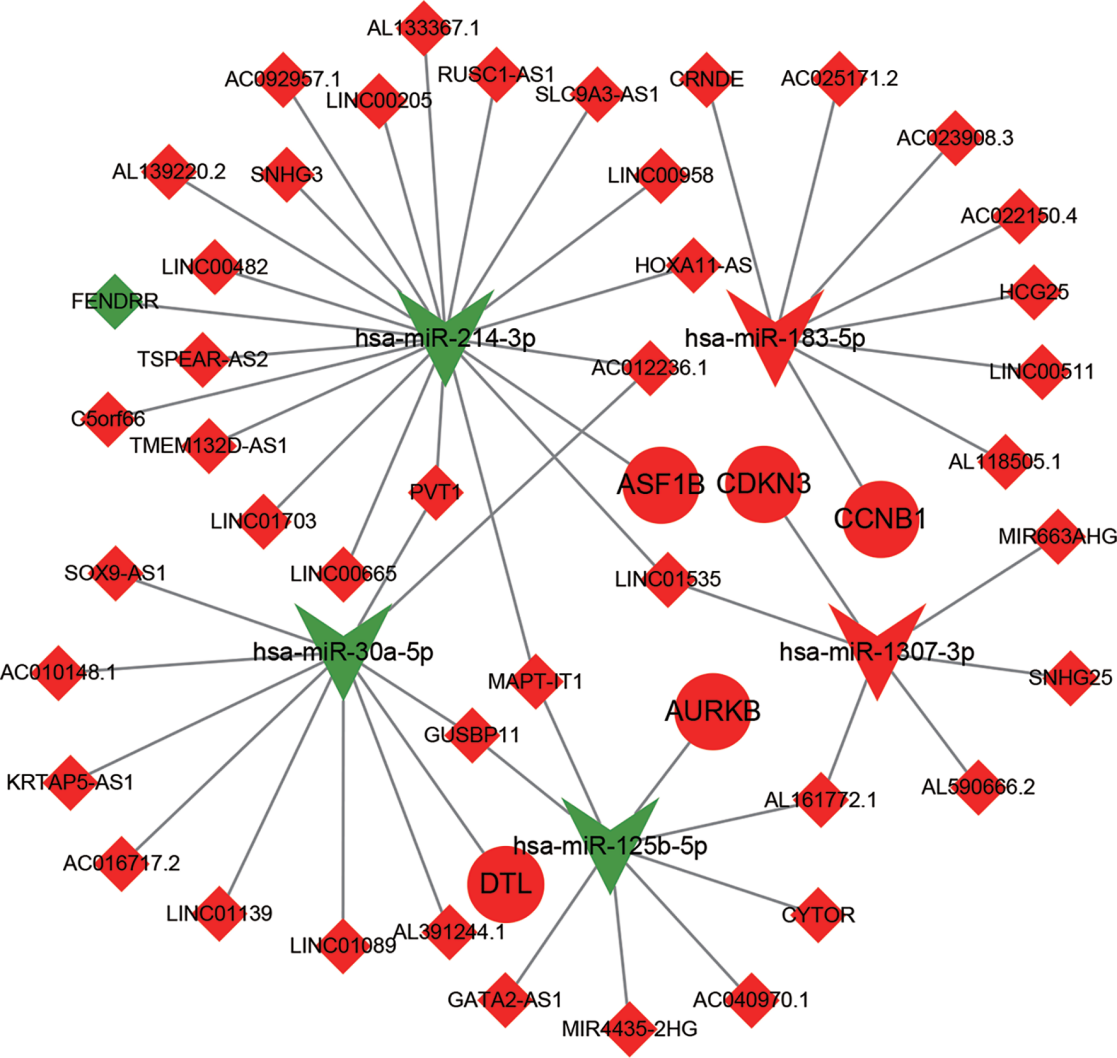
The construction of ceRNA sub-network based on relapse-related DEMs. Red represent up-regulated; green represent down-regulated; diamonds represent lncRNA; balls represent mRNA; triangle represent miRNA.

We first compared expression levels of hub genes between HCC patients with and without recurrence within 3 years. The results showed that the expression of *ASF1B* (P=0.023), *AURKB* (P=0.026), *CCNB1* (P=0.041), *CDKN3* (P=0.042), and *DTL* (P=0.015) was significantly higher in patients with recurrence compared with those without recurrence (**Figures 3A–E**). The relationships between these five mRNAs and HCC grade and stage were further analyzed. As shown in **Figures 3F–O**, higher expression levels of these mRNAs were significantly associated with higher tumor grade and more advanced tumor stage. A subsequent DFS analysis using GEPIA showed that HCC patients with high expression of *ASF1B*, *AURKB*, *CCNB1*, *CDKN3*, and *DTL* had poor DFS (**Figure 4**). These findings further indicated that *ASF1B*, *AURKB*, *CCNB1*, *CDKN3*, and *DTL* could be prognostic markers of HCC relapse. In addition, an lncRNA–miRNA–mRNA sub-network based on five relapse-related DEMs was constructed using Cytoscape (**Figure 5**).

### Identification of Relapse-Related DELs

According to the lncRNA–miRNA–mRNA sub-network, miR-183-5p, miR-1307-3p, and their corresponding lncRNAs were all upregulated in HCC; however, according to ceRNA theory, there should be a negative correlation between lncRNAs and miRNAs. Therefore, we removed these two miRNAs and their corresponding lncRNAs from the network, and the remaining lncRNAs were used to find relapse-related DELs.

**Figure 6:**
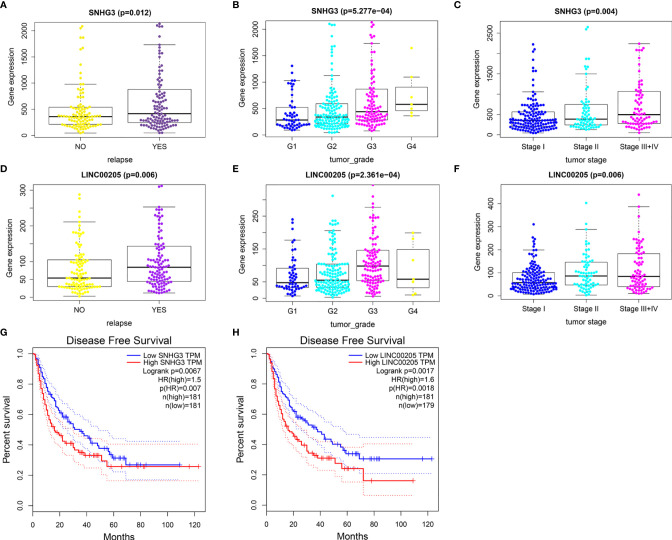
Identification of relapse-related DELs. **(A)** The relationship between SNHG3 and HCC recurrence. **(B)** The relationship between SNHG3 and tumor grade. **(C)** The relationship between SNHG3 and tumor stage. **(D)** The relationship between LINC00205 and HCC recurrence. **(E)** The relationship between LINC00205 and tumor grade. **(F)** The relationship between LINC00205 and tumor stage. **(G, H)** Disease-free survival analysis of SNHG3 and LINC00205 preformed using GEPIA.

The results showed that nine lncRNAs were highly expressed in HCC patients with relapse compared with those without relapse (**Figure S2** and **Figures 6A, D**), and the expression levels of *SNHG3* and *LINC00205* were closely correlated with HCC grade and stage (**Figures 6B, C, E, F**). Survival analysis further indicated that patients with high expression of *SNHG3* and *LINC00205* had shorter DFS than those with low expression (**Figures 6G, H**).

### Identification of Relapse-Related lncRNA–miRNA–mRNA Network

**Figure 7:**
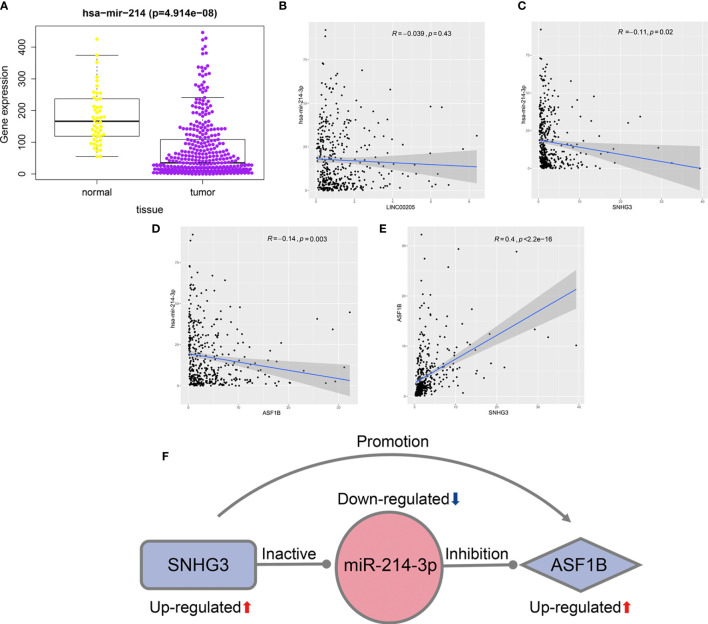
Identification of potential regulatory axis associated with HCC recurrence. **(A)** The relationship between the expression of miR-214-3p and HCC. **(B)** The correlation of LINC00205 and miR-214-3p. **(C)** The correlation of SNHG3 and miR-214-3p. **(D)** The correlation of ASF1B and miR-214-3p. **(E)** The correlation of SNHG3 and ASF1B. **(F)** A potential regulatory axis and the interactions among SNHG3, miR-214-3p and ASF1B.

As miR-214-3p is the common target of *SNHG3* and *LINC00205*, its expression levels in HCC tissues and normal liver tissues were also verified using TCGA-LIHC data. The results confirmed that it was significantly downregulated in HCC tissues (**Figure 7A**). Correlation analysis was performed to acquire results that were more reliable. The results showed that there was no correlation between LINC00205 and miR-214-3p (R=-0.039, P=0.43; **Figure 7B**). MiR-214-3p-ASF1B (R=-0.14, P=0.003) and SNHG3-miR-214-3p (R=-0.11, P=0.02) had negative correlations (**Figures 7C, D**), whereas ASF1B was significantly positively correlated with SNHG3 (R=0.4; P<2.2e-16; **Figure 7E**). Finally, a potential lncRNA–miRNA–mRNA regulatory axis associated with HCC recurrence was identified. The interactions among its members are shown in **Figure 7F**.

### Experimental Verification of SNHG3–miR-214-3p–ASF1B Regulatory Axis

**Figure 8:**
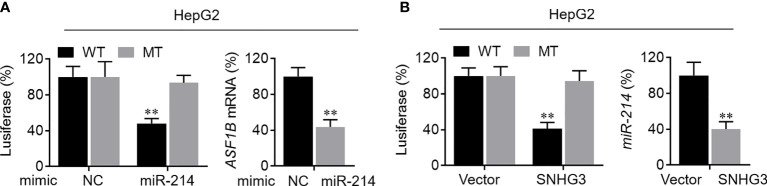
Verification of SNHG3-miR-213-3p-ASF1B regulatory axis. **(A)** Left: the regulatory relationship between ASF1B and miR-214-3p was validated in luciferase reporter assay; right: the mRNA level of ASF1B in HepG2 cells was markedly downregulated by miR-214-3p overexpression. **(B)** Left: the regulatory relationship between lncRNA SNHG3 and miR-214-3p was validated in luciferase reporter assay; right: the mRNA level of miR-214-3p in HepG2 cells was significantly decreased by SNHG3 overexpression. **p < 0.01.

First, the binding sites of miRNA-214-3p in the SNHG3 and ASF1B sequences were predicted using the starBase platform. To verify the regulatory effect of miR-214-3p on ASF1B, we constructed WT and MT sequences of ASF1B. ASF1B-WT and ASF1B-MT were co-transfected with miR-214-3p mimics into HepG2 cells. As expected, the dual-luciferase reporter gene assay results indicated that overexpression of miR-214-3p significantly reduced the luciferase activities of cells transfected with vectors containing ASF1B-WT but not ASF1B-MT (P<0.01), and expression of ASF1B was markedly downregulated by overexpression of miR-214-3p (P<0.01; **Figure 8A**). These results confirmed that miR-214-3p could directly bind to ASF1B and negatively regulate ASF1B expression. Next, vectors containing miR-214-3p-WT or miR-213-3p-MT were constructed and co-transfected with a SNHG3-overexpression plasmid into HepG2 cells. Luciferase activity was significantly inhibited (P<0.01) in cells containing the miR-214-3p-WT plasmid but not significantly altered in the miR-214-3p-MT group, whereas the expression level of miR-214-3p declined dramatically in HepG2 cells transfected with the SNHG3-overexpression plasmid (P<0.01; **Figure 8B**). The results of the dual-luciferase reporter gene assay suggested that lncRNA SNHG3 could directly sponge miR-214-3p. Moreover, SNHG3 could inhibit miR-214-3p expression.

### Correlation Analysis Between ASF1B and Tumor Immune Infiltration

**Figure 9:**
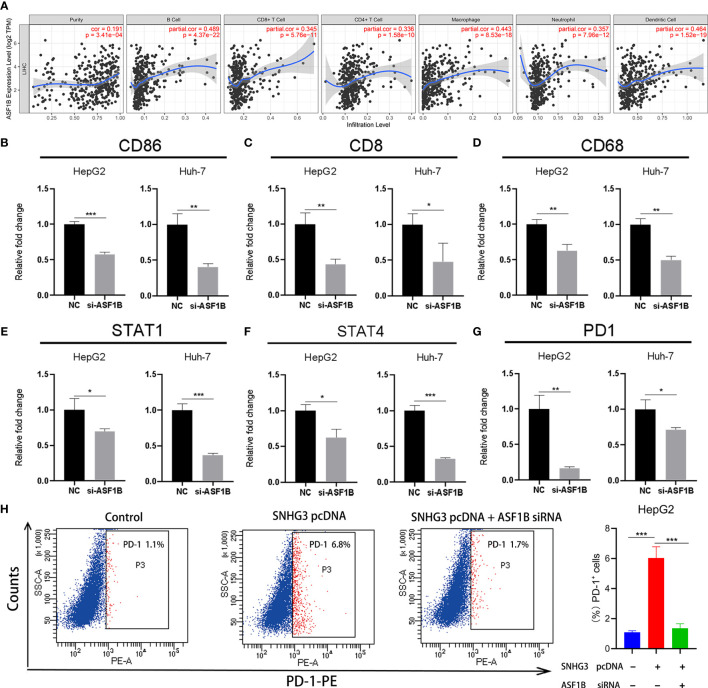
The relationship between ASF1B and tumor immune infiltration. **(A)** The expression of ASF1B was positively correlated with B cell, CD8+ T cell, CD4+ T cell, macrophage, neutrophil and dendritic cell immune infiltration levels in HCC. **(B–G)** The expression levels of CD86, CD8, CD68, STAT1, STAT4 and PD1 significantly reduced in HepG2 and Huh-7 cells by ASF1B knockdown. **(H)** HepG2 cell was transfected with SNHG3 pcDNA or si-ASF1B, and examined for the percentage of PD-1-positive cells. *p < 0.05, **p < 0.01, ***p < 0.001.

Notably, *ASF1B*, a key gene associated with HCC recurrence in this study, has not been previously reported in the HCC-related literature. Previous studies have confirmed that tumor infiltration is associated with the recurrence of HCC (8, 20). Therefore, we verified whether expression levels of *ASF1B* in HCC were correlated with immune infiltration, using the TIMER database. The results showed that expression levels of *ASF1B* were significantly positively correlated with immune infiltration of B cells (COR=0.489, p<0.001), CD8+ T cells (COR=0.345, p<0.001), CD4+ T cells (COR=0.336, p<0.001), macrophages (COR=0.442, p<0.001), neutrophils (COR=0.357, p<0.001), and dendritic cells (COR=0.464, p<0.001) in HCC (**Figure 9A**).

Subsequently, we further explored the relationships between the expression of *ASF1B* and gene markers of B cells, CD8+ T cells, neutrophils, dendritic cells, monocytes, Treg cells, macrophages, and natural killer cells in LIHC using TIMER. The results showed that *ASF1B* was positively correlated with *CD19* and *CD86* in B cells and with *CD8B* in CD8+ T cells. *ASF1B* in LIHC was also positively related to *SIGLEC5*, *KIR2DL4*, and *CSF3R* in neutrophils; *ITGAX*, *STAT4*, and *STAT1* in dendritic cells; *PD1* and *CCR8* in Treg cells; and *CD68* in monocytes (COR˃0.2). The results remained unchanged after adjustment for tumor purity and patient age (**Table 1**). As tumor purity has an important influence on immune infiltration levels (21), partial immune genetic markers with COR greater than 0.3 after tumor purity adjustment were selected, and RT-qPCR was used to verify whether their expression was affected by ASF1B. The results showed significantly decreased expression of CD86, CD8, STAT1, STAT4, CD68, and PD1 in HepG2 and Huh-7 cells following ASF1B knockdown (**Figures 9B–G**). These results were consistent with those of the bioinformatics analysis and further confirmed that *ASF1B* expression in HCC was closely related to immune infiltration. Therefore, we concluded that *ASF1B* affects recurrence partly *via* immune infiltration.

**Table 1:** Correlation analysis between ASF1B and immune cell type markers in TIMER database.

Cell type	Gene markers	LIHC					
		None		Purity		Age	
		COR	P	COR	P	COR	P
B cells	CD79A	0.156	2.54E-03	0.289	4.46E-08	0.139	7.67E-03
	CD19	0.269	1.36E-07	0.361	4.57E-12	0.251	1.18E-06
	CD86	0.271	1.17E-07	0.438	1.29E-17	0.271	1.47E-07
CD8+ T cells	CD8A	0.194	1.38E-04	0.323	7.60E-10	0.196	1.58E-04
	CD8B	0.201	9.72E-05	0.32	1.14E-09	0.203	9.51E-05
Neutrophils	CCR7	0.073	1.62E-01	0.23	1.61E-05	0.058	2.71E-01
	SIGLEC5	0.226	1.09E-05	0.366	2.23E-12	0.224	1.55E-05
	KIR2DL3	0.187	2.84E-04	0.238	7.85E-06	0.194	1.84E-04
	KIR2DL4	0.23	7.56E-06	0.271	3.09E-07	0.25	1.24E-06
	CSF3R	0.254	6.79E-07	0.403	6.93E-15	0.247	1.76E-06
	FPR1	0.183	3.99E-04	0.33	3.26E-10	0.187	3.22E-04
Dendritic cells	CD209	0.093	7.38E-02	0.181	7.16E-04	0.1	5.67E-02
	ITGAX	0.283	2.85E-08	0.425	1.37E-16	0.282	3.86E-08
	STAT4	0.242	2.24E-06	0.314	2.42E-09	0.23	9.15E-06
	STAT1	0.335	3.76E-11	0.387	8.70E-14	0.33	9.45E-11
Monocyte	CSF1R	0.142	6.23E-03	0.3	1.25E-08	0.146	5.24E-03
	CCL2	0.07	1.81E-01	0.195	2.74E-04	0.062	2.34E-01
	CD68	0.204	7.48E-05	0.309	4.66E-09	0.206	7.33E-05
Treg cells	FOXP3	0.147	4.57E-03	0.233	1.25E-05	0.172	9.75E-04
	PD1	0.315	5.63E-10	0.428	9.06E-17	0.304	2.92E-09
	CCR8	0.319	3.23E-10	0.422	2.66E-16	0.322	3.02E-10
Macrophages	PTGS2	0.056	2.84E-01	0.189	4.02E-04	0.038	4.66E-01
	CD163	0.039	4.51E-01	0.16	2.93E-03	0.045	3.93E-01
	MS4A4A	0.057	2.72E-01	0.2	1.79E-04	0.06	2.52E-01
	CEACAM8	0.054	2.97E-01	0.088	1.01E-01	0.049	3.53E-01
Natural killer cells	KIR3DL3	0.045	3.84E-01	0.053	3.23E-01	0.038	4.72E-01
	KIR2DS4	0.078	1.33E-01	0.077	1.51E-01	0.072	1.67E-01

COR, r value of Spearman’s correlation; Purity, correlation adjusted by purity; Age, correlation adjusted by age.

In addition, as an important target of immunotherapy, PD-1 was closely related to ASF1B in our study. To further validate the role of SNHG3/miR-214-3p/ASF1B axis on PD-1, HCC cell was transfected with SNHG3 overexpression plasmid and si-ASF1B. The result of flow cytometry showed that PD-1 was significantly upregulated after transfection of SNHG3 overexpression plasmid (p<0.001), and ASF1B knockdown partially reversed the effect of lncRNA SNHG3 on PD-1 expression in HCC cell (p<0.001) (**Figure 9H**). Thus, these data indicated that lncRNA SNHG3 promoted the expression level of PD-1 by regulating ASF1B in HCC.

**Figure 10:**
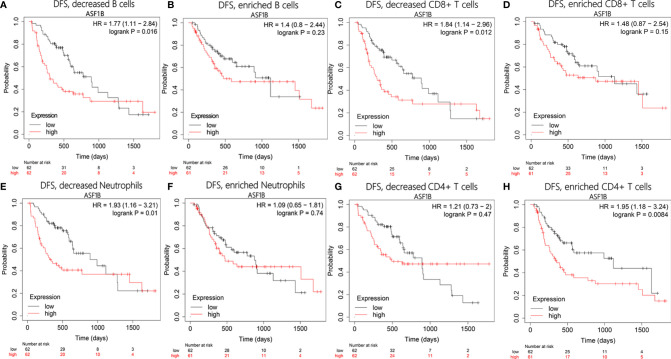
Disease-free survival analysis of ASF1B in following immune cells infiltration subgroups: **(A, B)** B cells, **(C, D)** CD8+ T cells, **(E, F)** Neutrophils, **(G, H)** CD4+ T cells.

To test whether there was an interaction between immune infiltration and *ASF1B*, we performed a prognostic analysis based on the expression levels of *ASF1B* in different immune cell subgroups using Kaplan–Meier plotter. The results showed that HCC patients with high expression of *ASF1B* had higher recurrence rates in the decreased B cells [hazard ratio (HR)=1.77, P=0.016], decreased CD8+ T cells (HR=1.84, P=0.012), and decreased neutrophils (HR=1.93, P=0.01) subgroups (**Figures 10A, C, E**). However, there was no significant correlation between high *ASF1B* expression and recurrence in subgroups enriched in these immune cells (**Figures 10B, D, F**). On the contrary, high expression of *ASF1B* was closely associated with worse DFS in the enriched CD4+ T cells subgroup (HR=1.95, P=0.0084; **Figure 10H**), but there was no such correlation in the decreased CD4+ T cells cohort (HR=1.21, P=0.47; **Figure 10G**). These results indicate that high *ASF1B* expression may influence HCC recurrence partly owing to immune infiltration.

## Discussion

Liver cancer is the second leading cause of tumor-related deaths globally, especially in developing countries (1). Owing to a limited organ supply, surgery remains the most important treatment strategy. Unfortunately, the rate of HCC recurrence after surgical treatment is high, and no effective solution is available to reduce the risk of recurrence. Therefore, there is an urgent need to identify new biomarkers to predict recurrence, and to improve our understanding of the mechanism of tumor recurrence. The rapid development of high-throughput sequencing technologies and improvements in bioinformatics methods enable us to explore the genetic changes in HCC.

In this research, we used bioinformatics and integrated analyses of multiple databases to construct a preliminary ceRNA network consisting of 124 DELs, 35 DEMIs, and 80 DEMs. Subsequently, we established a PPI network based on these DEMs to screen hub mRNAs for further analysis. Five of the 22 hub mRNAs (*ASF1B*, *AURKB*, *CCNB1*, *CDKN3*, and *DTL*) were found to be significantly overexpressed in HCC tissues of patients with recurrence within 3 years. Moreover, the expression levels of these five mRNAs were also significantly associated with tumor grade and stage. DFS analysis showed that the five mRNAs were closely related to poor prognosis of patients with HCC. The results for *AURKB* in our study were consistent with those of Lin et al. (22), who reported that *AURKB* was overexpressed in tumoral specimens and associated with early recurrence and poor prognosis after surgery. A previous study reported that the upregulation of *CCNB1* was closely associated with worse overall survival and DFS (23). This conclusion was further confirmed by the finding that knockdown of *CCNB1* inhibited cell proliferation, migration, and invasion in HCC (24). Zhou et al. (25) found by bioinformatics analysis that *CCNB1*, *DTL*, and *CDKN3* were upregulated in HCC tissue and related to lower survival rates, consistent with our results. Moreover, we also found that *CCNB1*, *DTL*, and *CDKN3* were closely related to recurrence in HCC.

Subsequently, a ceRNA sub-network was screened based on the five relapse-related mRNAs. Two lncRNAs (*SNHG3* and *LINC00205*) were significantly highly expressed in HCC patients with recurrence. *SNHG3* has been widely reported to have a role in prognosis of HCC patients and to be overexpressed in HCC tissues (26–28). Moreover, *SNHG3* expression was significantly higher in highly metastatic HCC cells compared with less-metastatic HCC cells, and overexpression of *SNHG3* promoted cell invasion, epithelial–mesenchymal transition, and sorafenib resistance in HCC (29). The results for *SNHG3* in our study are consistent with those of previous reports. We also found that high expression of *SNHG3* was related to poor DFS and indicated higher tumor grade and more advanced tumor stage. Studies in other cancers have reported similar results; high expression of *SNHG3* was found to be significantly associated with poor prognosis in gastric cancer (30), osteosarcoma (31), laryngeal carcinoma (32), non-small-cell lung cancer (33), and colorectal cancer (34). Overexpression of *LINC00205* has been shown to be closely associated with poor prognosis in HCC (35) and to promote cell proliferation, migration, and invasion by targeting miR-122-5p (36). These results further confirm that lncRNAs *SNHG3* and *LINC00205* have important roles in HCC.

Correlation analysis was used to identify a regulatory axis involving five mRNAs and two lncRNAs related with relapse. Finally, *SNHG3*–*miR-214-3p*–*ASF1B* was shown to be a potential regulatory axis associated with HCC recurrence; this result was further verified by the dual-luciferase reporter gene assay. A recent study reported that miR-214-3p was downregulated in HCC tissues (37), consistent with our results. The expression of miR-214-3p was also shown to be closely correlated with recurrence and survival of liver transplant patients, and miR-214-3p was found to inhibit HCC proliferation by targeting *MELK* (38). In addition, previous reports confirmed that miR-214-3p could inhibit the progression of multiple myeloma by targeting *ASF1B* (39), as well as being regulated by lncRNA *SNHG3* to affect the development of papillary thyroid carcinoma (40). These findings provide further confirmation that *SNHG3* regulates the expression of *ASF1B* by targeting miR-214-3p.

Extensive studies have reported that *ASF1B* is significantly upregulated in tumor tissues and promotes progression of various cancers, including prostate cancer, cervical cancer, and clear cell renal cell carcinoma (41–43), but there has been no previous report of its role in HCC. Here, we first showed that *ASF1B* has potential as a prognostic marker of recurrence in HCC. We also found that the expression of *ASF1B* was strongly positively correlated with infiltration levels of B cells, CD8+ T cells, CD4+ T cells, macrophages, neutrophils, and dendritic cells in HCC. Furthermore, there were significant correlations between the expression levels of *ASF1B* and most gene markers of B cells, CD8+ T cells, neutrophils, dendritic cells, monocytes, and Treg cells. Our results also confirm that reducing ASF1B could significantly inhibit the expression of CD86, CD8, STAT1, STAT4, CD68, and PD1. In recent decades, convincing evidence has shown that the tumor microenvironment plays an important part in the development of many malignant tumors (44). HCC patients with early relapse have been reported to have increased levels of dendritic cells and CD8+ T cells; this change may result in the activation of immune evasion mechanisms associated with tumor relapse (45). PD1 has been reported to inhibit the immune response of HCC patients by negatively regulating the activation and function of T cells, thereby contributing to tumor aggressiveness and postoperative recurrence (20). Our study also further confirmed that lncRNA SNHG3 promoted the expression level of PD-1 by regulating ASF1B in HCC. This evidence strongly suggests that SNHG3/miR-214-3p/ASF1B axis promotes HCC recurrence by activating tumor immune tolerance and escape, and PD-1 plays an crucial role in this process. In addition, the prognostic analysis showed that the high *ASF1B* expression group had worse DFS and higher HR in the decreased B cells, decreased CD8+ T cells, decreased neutrophils and enriched CD4+ T cells subgroups. CD4+ T cells have been reported to inhibit proliferation of effector T cells and to promote the evasion of HCC cells of the anti-tumor immune response (46). Therefore, *ASF1B* is expected to be a new target of immunotherapy for HCC.

In summary, in this study we constructed a novel lncRNA–miRNA–mRNA network associated with recurrence in HCC and found that lncRNA SNHG3 could promote the recurrence of HCC by regulating ASF1B expression *via* sponging of miR-214-3p. Furthermore, our results indicate that *ASF1B* is related to immune cell infiltration and recurrence in HCC, and suggest a new immunotherapy-based strategy to reduce the risk of recurrence after surgery.

## Data Availability Statement

The datasets presented in this study can be found in online repositories. The names of the repository/repositories and accession number(s) can be found in the article/**Supplementary Material**.

## Ethics Statement

Our research conforms completely to the TCGA and GEO publication requirement, and the approval of the ethics committee was not needed.

## Author Contributions

TZ and XG analyzed the data, performed experiments and wrote the manuscript. GW and FL collected data and helped perform the analysis with constructive discussions. JS, CL, and LX revised the manuscript. JZ and YL conceived and directed the research. All authors contributed to the article and approved the submitted version.

## Funding

This work was supported by the Natural Science Foundation of China (81874058 and 81961160708), and the Major Projects of Science and Technology Development Fund of Nanjing Medical University (NMUD2019008).

## Conflict of Interest

The authors declare that the research was conducted in the absence of any commercial or financial relationships that could be construed as a potential conflict of interest.
