# Supplementary material for: Construction of Novel lncRNA–miRNA–mRNA Network Associated With Recurrence and Identification of Immune-Related Potential Regulatory Axis in Hepatocellular Carcinoma
Source: Front Oncol. 2021 Jul 15;11:626663. doi: 10.3389/fonc.2021.626663 (PMC8320021; doi:10.3389/fonc.2021.626663)
Supplement: Supplementary file 1 [file DataSheet_1.docx]

Supplementary Material

## Supplementary Tables

**Table S1 The primer sequences in our study.**

| Gene | Sequence (5' -> 3') |
| --- | --- |
| ASF1B Forward Primer | TCCGGTTCGAGATCAGCTTC |
| ASF1B Reverse Primer | GTCGGCCTGAAAGACAAACA |
| SNHG3 Forward Primer | TTCAAGCGATTCTCGTGCC |
| SNHG3 Reverse Primer | AAGATTGTCAAACCCTCCCTGT |
| miR-214-3p Forward Primer | AGTGCAGGGTCCGAGGTATT |
| miR-214-3p Reverse Primer | GCGACAGCAGGCACAGACA |
| GAPDH Forward Primer | ACAACTTTGGTATCGTGGAAGG |
| GAPDH Reverse Primer | GCCATCACGCCACAGTTTC |
| CD86 Forward Primer | CTGCTCATCTATACACGGTTACC |
| CD86 Reverse Primer | GGAAACGTCGTACAGTTCTGTG |
| CD8 Forward Primer | ATGGCCTTACCAGTGACCG |
| CD8 Reverse Primer | AGGTTCCAGGTCCGATCCAG |
| STAT1 Forward Primer | CAGCTTGACTCAAAATTCCTGGA |
| STAT1 Reverse Primer | TGAAGATTACGCTTGCTTTTCCT |
| STAT4 Forward Primer | TGTTGGCCCAATGGATTGAAA |
| STAT4 Reverse Primer | GGAAACACGACCTAACTGTTCAT |
| CD68 Forward Primer | CTTCTCTCATTCCCCTATGGACA |
| CD68 Reverse Primer | GAAGGACACATTGTACTCCACC |
| PD1 Forward Primer | CCAGGATGGTTCTTAGACTCCC |
| PD1 Reverse Primer | TTTAGCACGAAGCTCTCCGAT |

## Supplementary Figures


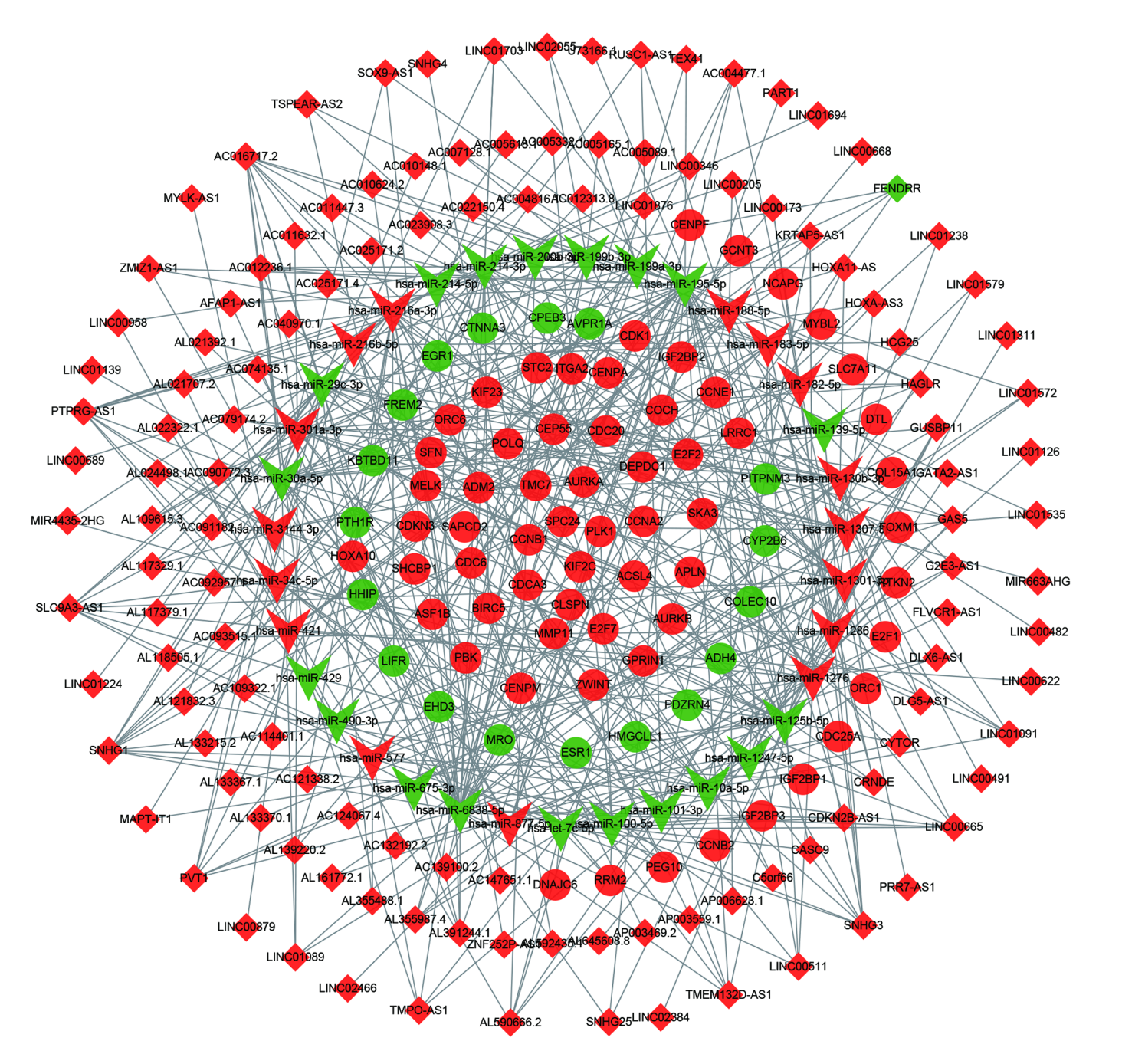


**Figure S1.** The construction of a preliminary ceRNA network.


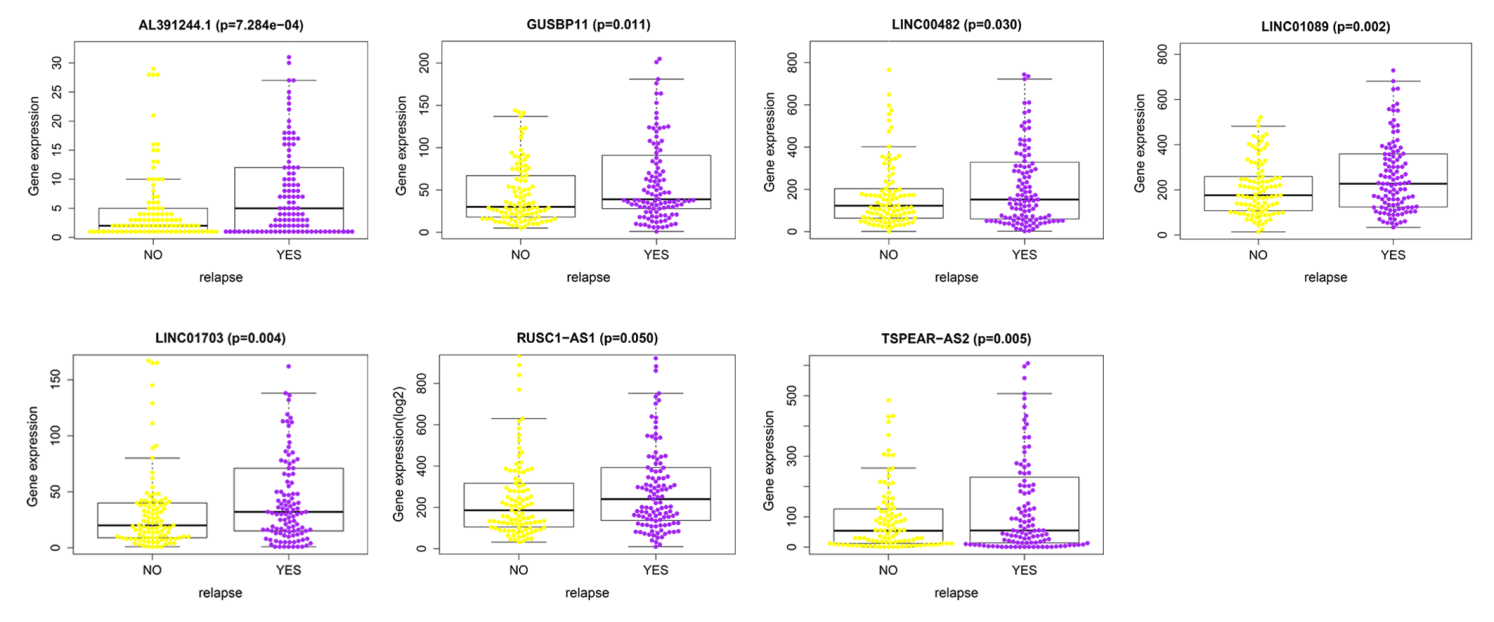


**Figure S2** The expressions of AL391244.1, GUSBP11, LINC00482, LINC01089, LINC01703, RUSC1-AS1 and TSPEAR-AS1 were significantly associated with HCC recurrence.

## Supplementary Tables

**Table S2 The primer sequences.**

| Gene | Sequence (5' -> 3') |
| --- | --- |
| ASF1B Forward Primer | TCCGGTTCGAGATCAGCTTC |
| ASF1B Reverse Primer | GTCGGCCTGAAAGACAAACA |
| SNHG3 Forward Primer | TTCAAGCGATTCTCGTGCC |
| SNHG3 Reverse Primer | AAGATTGTCAAACCCTCCCTGT |
| miR-214-3p Forward Primer | AGTGCAGGGTCCGAGGTATT |
| miR-214-3p Reverse Primer | GCGACAGCAGGCACAGACA |
| GAPDH Forward Primer | ACAACTTTGGTATCGTGGAAGG |
| GAPDH Reverse Primer | GCCATCACGCCACAGTTTC |
| CD86 Forward Primer | CTGCTCATCTATACACGGTTACC |
| CD86 Reverse Primer | GGAAACGTCGTACAGTTCTGTG |
| CD8 Forward Primer | ATGGCCTTACCAGTGACCG |
| CD8 Reverse Primer | AGGTTCCAGGTCCGATCCAG |
| STAT1 Forward Primer | CAGCTTGACTCAAAATTCCTGGA |
| STAT1 Reverse Primer | TGAAGATTACGCTTGCTTTTCCT |
| STAT4 Forward Primer | TGTTGGCCCAATGGATTGAAA |
| STAT4 Reverse Primer | GGAAACACGACCTAACTGTTCAT |
| CD68 Forward Primer | CTTCTCTCATTCCCCTATGGACA |
| CD68 Reverse Primer | GAAGGACACATTGTACTCCACC |
| PD1 Forward Primer | CCAGGATGGTTCTTAGACTCCC |
| PD1 Reverse Primer | TTTAGCACGAAGCTCTCCGAT |
